# MicroRNA-195 suppresses tumor cell proliferation and metastasis by directly targeting BCOX1 in prostate carcinoma

**DOI:** 10.1186/s13046-015-0209-7

**Published:** 2015-09-04

**Authors:** Jia Guo, Min Wang, Xiuheng Liu

**Affiliations:** Department of Urology, Renmin Hospital of Wuhan University, Wuhan University, Jiefang Road 238, Wuhan, 430060 Hubei People’s Republic of China

**Keywords:** miRNA, Metastasis, miR-195, Prostate cancer, BCOX1

## Abstract

Elucidation of the downstream targets regulated by the metastasis-suppressive miRNAs can shed light on the metastatic processes in prostate cancer (PCa). We conducted microarray analyses and found that miR-195 was significantly decreased in metastatic PCa. Low miR-195 expression is an independent prognostic factor for poor biochemical recurrence-free and overall survival. Forced expression of miR-195 in PCa cells drastically inhibits proliferation, migration and invasion *in vitro* and inhibits tumor growth and metastasis *in vivo*. BCOX1 is identified as a direct target of miR-195 in PCa, and is found to be drastically increased in metastatic PCa. BCOX1 knockdown phenotypically copies miR-195-induced phenotypes, whereas forced expression of BCOX1 reverses the effects of miR-195. Collectively, this is the first report unveils that loss of miR-195 expression and thus uncontrolled BCOX1 upregulation might drive PCa metastasis.

**Electronic supplementary material:**

The online version of this article (doi:10.1186/s13046-015-0209-7) contains supplementary material, which is available to authorized users.

## Introduction

Prostate cancer (PCa) is the most commonly diagnosed non-cutaneous malignancy worldwide among men and the second most common cause of male cancer-related deaths [1]. Despite great improvement in the early diagnosis and treatment options, the outcome of some PCa patients remains unsatisfactory, mainly because of cancer recurrence and metastasis. PCa patients are generally androgen-sensitive at the initial diagnosis. However, patients eventually develop metastatic androgen-independent PCa. PCa metastasis is multistage processes that involve lots of oncogenes and tumor suppressor genes. Therefore, it is urgent to find effective biomarkers to strengthen the efficiency of early diagnosis and to improve the therapeutic strategies of PCa. A better understanding of the molecular events underlying the PCa metastasis is very important for its prevention, diagnosis and treatment.

Accelerating evidence links miRNAs to the initiation, development, promotion, and progression of malignancies [2]. miRNAs are highly conserved small non-coding regulatory RNAs with sizes of 17–25 nucleotides [3, 4]. More than 50 % of the known miRNAs were involved in tumorigenesis and/or metastasis by directly targeting molecular targets [5]. Although the importance of miRNAs in metastasis has attracted much attention in recent years, the pathological relevance and significance of the majority of miRNAs in PCa remain unclear.

To the best of our knowledge, the molecular mechanism of miR-195 deregulation in PCa remain elusive, so our study aimed to investigate the biological functions and underlying molecular mechanisms of miR-195 in PCa. We firstly conducted microarray analyses and found miR-195 as a metastasis associated miRNA, and then we found significant associations between miR-195 expression and the clinicopathological factors and prognosis of PCa patients. We further explored its effects on the malignant phenotype of PCa cells. We found that the miR-195 can regulate the invasiveness of PCa cells and the metastasis of PCa xenografts by regulating BCOX1. Collectively, above findings have advanced our knowledge of the molecular mechanisms of PCa metastasis, provided potential effective molecular biomarkers for the diagnosis and prognosis, and developed effective potential therapeutic targets for the treatment of PCa.

## Materials and methods

### Patients

140 PCa and paired adjacent normal tissues were obtained from the Renmin hospital of Wuhan university, from patients who underwent radical prostatectomy between 2002 and 2009. None of the patients had received androgen deprivation treatment, chemotherapy or radiation therapy prior to the surgery. Informed consent was obtained from all patients. This study was approved by the research ethics committee of our hospital (WHRMh-2014029). This investigation conformed to the principles outlined in the Declaration of Helsinki.

### Cell lines

PCa cell lines PC-3 and LNCaP were grown in RPMI 1640 (Life Technologies, CA) with 0.023 IU/ml insulin and 10 % FBS (Invitrogen) in 5 % CO_2_ cell culture incubator.

### Plasmids and cell transfection

A cDNA sequence containing one pre-miR-195 unit was inserted into pcDNA3.1 (Promega, Madison, WI, USA). The BCOX1 shRNA was designed with a shRNA designer tool (http://rnaidesigner.thermofisher.com/rnaiexpress/). Two strands were annealed, followed by insertion into pcDNA6.2-GW/EmGFP-miR vector. The CTHRC1 cDNA containing the coding sequence was cloned by PCR, and the PCR product was cloned into the pcDNA3.1 vector. The insert was confirmed by DNA sequencing.

### Colony formation assay

In colony formation assay, the cells were seeded on 35-mm dishes. The cells were fixed in methanol, and then stained with crystal. Finally, positive colony formation (>50 cells/colony) was counted.

### Cell migration and invasion assay

Transwell migration and invasion assays were performed with 8.0-mm pore according to the manufacturers’ instructions (BD Bioscience, CA). The PCa cell migration and invasion assays were performed with uncoated (migration) and coated Matrigel (invasion). The migrated and invaded PCa cells in the membrane were fixed and stained, and the cells were counted under a microscope.

### Luciferase reporter assay

In brief, the miR-195-binding site in the BCOX1 3’-UTR region (wild or mutant-type) was cloned downstream of the firefly luciferase gene in a pGL3-promoter vector. The luciferase assay was performed following the manufacturer’s protocol. Luciferase activity was measured using the dual luciferase reporter assay system (Promega, Madison, WI).

### RNA extraction and qRT-PCR analyses

Total RNA was extracted using Trizol Reagent according to the manufacturer’s protocol. The expression level of miR-195 was measured by TaqMan miRNA assays (Applied Biosystems, CA, USA) according to the provided protocol, miRNA U6 was used for normalization. BCOX1 expression was measured by SYBR green qPCR assay and β-actin was used as an endogenous control.

### Western blot analysis

Protein concentration was measured by use of the BCA reagent kit (Merck). The protein was resolved by SDS-PAGE and transferred to a PVDF membrane, which was probed with specific primary antibody against BCOX1 (1:200). β-actin was blotted to show equal protein loading.

### Immunohistochemistry

After deparaffinization and rehydration, the tissues were washed by phosphate-buffered saline and treated with 3 % H_2_O_2_ in methanol for 10 min. After being washed with distilled water, the tissues were subjected to antigen retrieval in citrate buffer and stained overnight with rabbit polyclonal anti-BCOX1 antibody (Biorbyt, USA). The sections were incubated with goat anti-rabbit IgG for 30 min and developed with diaminobenzadine.

BCOX1 protein level was classified semiquantitatively combining the proportion and intensity of positively stained immunoreactive cells [6, 7]. The percentage of positive-staining cells was scored as follows: 0 (<5 % positive cells), 1 (5–50 % positive cells), and 2 (>50 % positive cells). Staining intensity was scored as follows: 0 (no staining or only weak staining); 1 (moderate staining); and 2 (strong staining). The sum of the staining intensity score and the percentage score was used to define the NUCB2 protein expression levels: 0–2, low expression and 3–4, high expression.

### Prostate tumor xenograft studies

We established xenografts in nude mice with the stable expressing miR-195 cells, BCOX1 + miR-195 (BCOX1 plus miR-195) cells, and control cells. PCa cells were implanted into the dorsal flank of male Athymic nude mice subcutaneously. Tumor size was measured biweekly, and tumor volumes were calculated using the formula: Volume (mm^3^) = [width^2^ (mm^2^) × length (mm)]/2. Mice with tumors were killed 7 weeks after the inoculation. The xenograft tumors, and the cervical lymph nodes were collected and tumor weights were measured. DNA extraction of the cervical lymph nodes and human alu sequence PCR amplification were performed as described previously [8].

### Statistical analysis

For continuous variables, Student’s t-test was performed. Spearman correlation test was chosen for examining the correlations between miR-195 expression level and the clinical and pathological variables. Survival curves were carried out by the Kaplan-Meier method and evaluated using the log-rank test. Identified factors were associated with survival by the Cox proportional hazard regression model. *P* < 0.05 was considered statistically significant. Statistical analysis was performed using SPSS 17.0 software.

## Results

### miR-195 is a potential anti-metastasis miRNA

We performed miRNA microarray data analyses from paired metastatic LTL-313H and non-metastatic LTL-313B PCa xenografts [9], and identified that miR-195 showed a 4.8-fold decrease in the metastatic line. We also performed clinical PCa miRNA microarray analyses and found that miR-195 is significantly decreased in metastatic tissues compared with primary PCa tissues (Additional file 1: Figure S1) [10].

### Correlations of miR-195 expression with clinicopathologic characteristics and prognosis of PCa

The association between miR-195 expression and clinicopathological factors was investigated in 140 PCa patients (Additional file 2: Table S1). The miR-195 expression in PCa tissues was divided in the low and high by the median value. The data indicated low miR-195 expression was significantly associated with lymph node metastasis, BCR, Gleason score, preoperative PSA, and seminal vesicle invasion (Additional file 2: Table S1). PCa patients with high miR-195 expression had better overall survival and BCR-free survival than patients with low miR-195 expression. Univariate Cox analysis showed that low miR-195 expression may affect both the BCR-free survival and overall survival of patients with PCa. Multivariate Cox regression analysis further confirmed that low miR-195 expression in PCa was an independent prognostic factor for poor overall survival and BCR-free survival (Additional file 3: Table S2 and Additional file 4: Table S3). Taken together, above results indicated that patients with low miR-195 expression tend to have a poorer prognosis.

### miR-195 inhibits the proliferation, migration and invasion of PCa cell lines

**Fig. 1:**
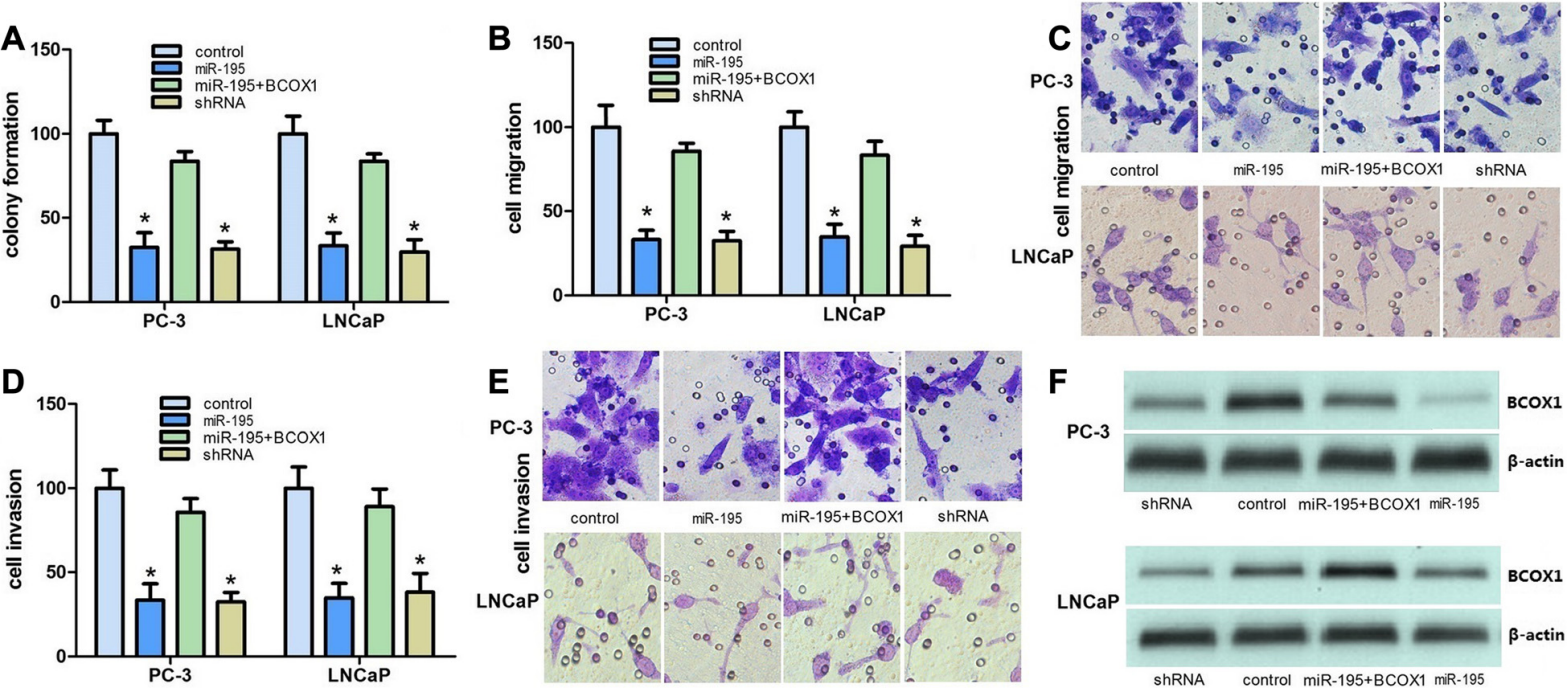
miR-195 inhibits PCa cell proliferation, migration and invasion *in vitro*. **a**, BCOX1 knockdown can mimic the suppression of colony formation induced by miR-195 in PC-3 and LNCaP cells. **b** and **c**, BCOX1 knockdown can mimic the suppression of migration activity induced by miR-195 in PC-3 and LNCaP cells. **d** and **e**, BCOX1 knockdown can mimic the suppression of invasion activity induced by miR-195 in PC-3 and LNCaP cells. **f**, The efficiency of BCOX1 knockdown and ectopic expression of miR-195 was confirmed at protein level by western blot. All data are shown as mean ± SD

To investigate the biological functions of miR-195 during PCa progression, we restored the expression of miR-195 in the PC-3 and LNCaP cells. Colony formation assays were performed to assess the role of miR-195 in PCa. We found that forced expression of miR-195 significantly inhibited colony formation compared to control cells (Fig. 1a). We further investigated whether miR-195 could affect migration and invasion of PCa cells. The results indicated that forced expression of miR-195 significantly reduced the invasion and migration of PCa cells (Fig. 1b, c, d, e). BCOX1 protein expression in PCa cells was studied by immunoblot analysis using BCOX1 antibody (Fig. 1f). Collectively, these results showed the downregulation of miR-195 can promote PCa progression *in vitro* by improving proliferation, invasion and migration.

### BCOX1 is a direct downstream target of miR-195

**Fig. 2:**
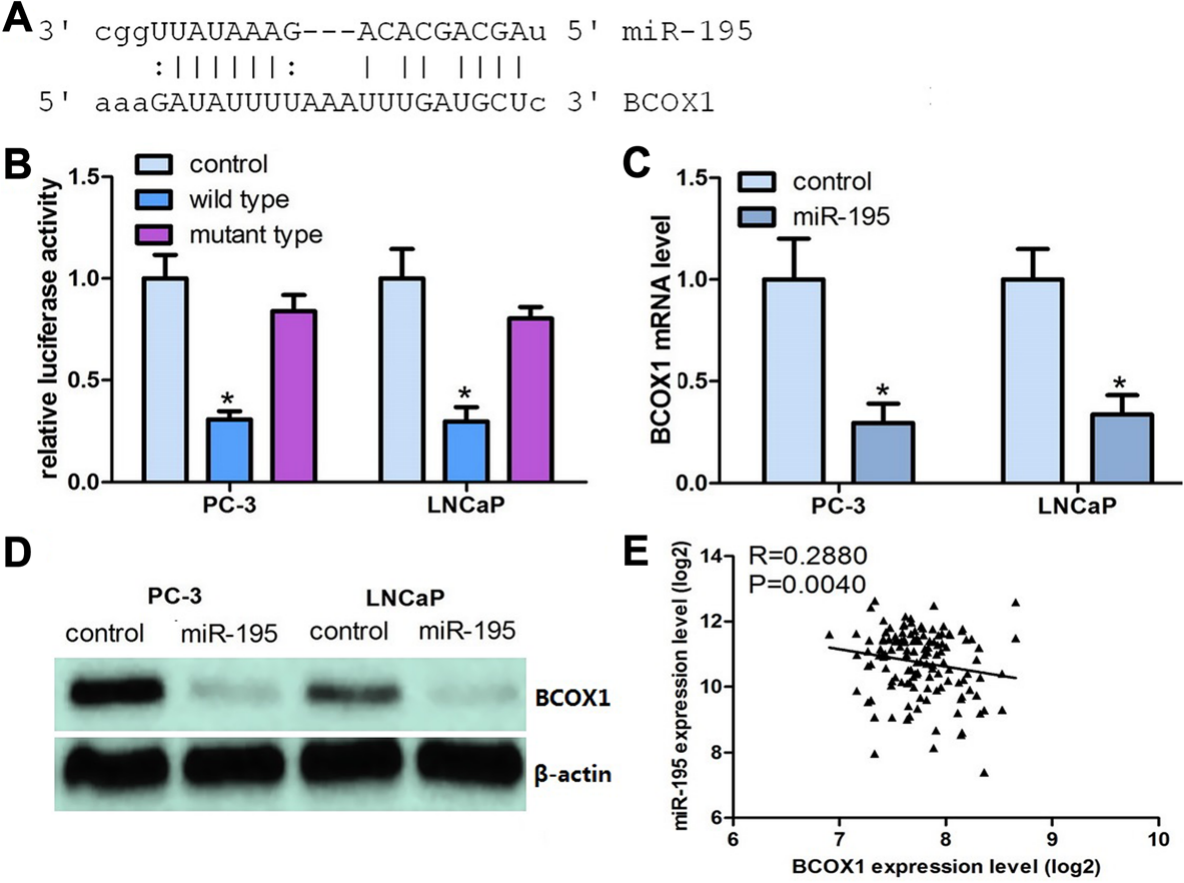
BCOX1 is a direct target of miR-195 in PCa tissues. **a** Computational analysis indicating that miR-195 potentially targeted BCOX1. **b** Relative luciferase activities were studied in PCa cells. **c** Decrease in BCOX1 mRNA expression by miR-195 was investigated using qRT-PCR. **d** Decrease in BCOX1-35 protein by miR-195 was studied using western blots. **e** BCOX1 mRNA was inversely associated with miR-195 in 140 pairs of PCa tissues using linear regression models

We investigated the candidate targets for miR-195 using prediction algorithm provided by miRanda. We selected BCOX1 for further validation due to its potential role in metastasis by microarray data analyses. Through computational analysis, the binding site for miR-195 at 3’-UTR of BCOX1 was depicted (Fig. 2a). We then carried out a luciferase-based assay to validate whether this gene was regulated by miR-195. Luciferase vectors containing the 3’-UTR of the gene were created and transfected along with or without the miR-195 expressing plasmid into PCa cells. The results indicated that co-transfection with miR-195 in PC-3 and LNCaP cells significantly decreased luciferase activity when the construct contained the 3’UTR of BCOX1 (Fig. 2b). Mutation of the binding sites can reverse the inhibitory effects.

qRT-PCR and western blot results confirmed the mRNA and protein expression levels of BCOX1 were significantly inhibited in miR-195 transfectants as compared with control groups (Fig. 2c, d). Moreover, BCOX1 mRNA levels were inversely correlated with the miR-195 expression levels in PCa tissues (Fig. 2e). Taken together, our results indicated that miR-195 can negatively regulate BCOX1 expression by directly binding to its 3’UTR.

### BCOX1 as a potential metastasis-associated gene in PCa by microarray data analyses

**Fig. 3:**
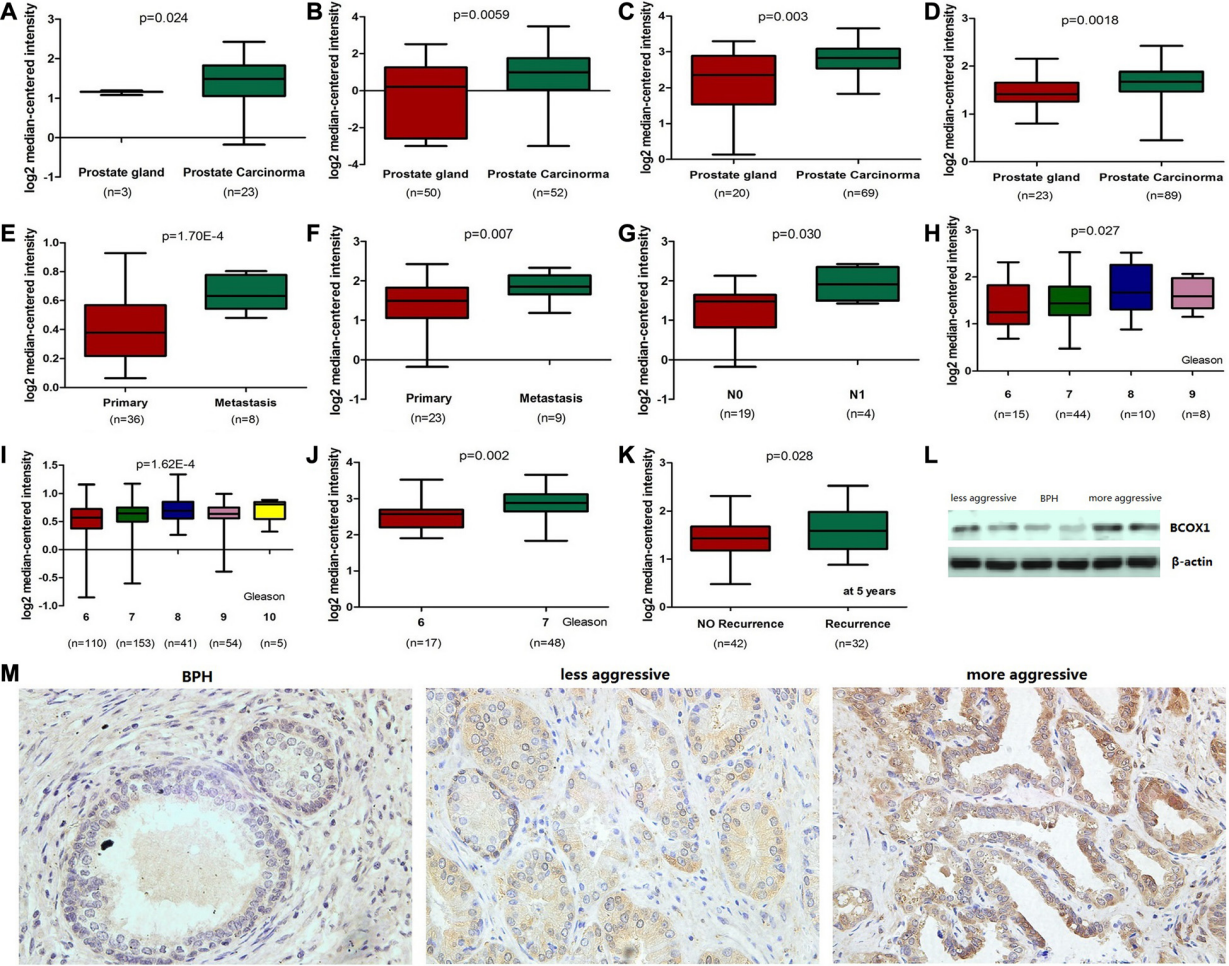
BCOX1 is upregulated in PCa tissues and is correlated with PCa progression. **a**, **b**, **c** and **d**, **e**, **f**, Box plots represent BCOX1 mRNA level in normal prostate, primary PCa and metastatic PCa tissues. **g**, **h**, **i**, **j** and **k**, BCOX1 is significantly upregulated in patients with N1, higher Gleason score, and BCR compared with patients with N0, lower Gleason score, and without BCR, respectively. **l**, BCOX1 protein expression by western blots of prostate tissues using BCOX1 antibody. **m**, Immunohistochemical analysis of BCOX1 in benign prostate epithelia, less aggressivePCa and more aggressive PCa

In order to investigate whether any significant difference of BCOX1 mRNA level exists in metastatic PCa, primary PCa and normal prostate tissues, several available datasets were analyzed [11–18]. The data indicated that BCOX1 mRNA was significantly increased in primary PCa tissues relative to normal prostate tissues (Fig. 3a, b, c, d) [11–14]. Similarly, increased BCOX1 mRNA was found in metastatic PCa relative to primary PCa tissues (Fig. 3e, f) [11, 15]. Microarray data indicated that BCOX1 is significantly increased in patients with lymph node metastasis, higher Gleason score and BCR compared with patients without lymph node metastasis, BCR, and with lower Gleason score, respectively (Fig. 3g, h, i, j, k) [11, 16–18].

To confirm these results, we conducted qRT-PCR using RNA from PCa and normal prostate samples. qRT-PCR analysis confirmed the overexpression of BCOX1 mRNA in PCa tissues compared with normal tissues as did western blots using BCOX1 antibody (Fig. 2l). Immunohistochemical analysis indicated weak or no reactivity in benign prostate samples but strong staining in the aggressive PCa tissues (Fig. 2m). Collectively, above data indicated BCOX1 is increased in PCa tissues and is associated with PCa progression.

### miR-195 inhibits cell proliferation, invasion and migration via the suppression of BCOX1

To determine whether miR-195-dependent inhibition of PCa cell proliferation migration, and invasion was indeed mediated by BCOX1, we used a complementary approach of gain- and loss-of function of BCOX1. BCOX1 was restored in PCa cells. The results of the colony formation indicated that forced expression of BCOX1 significantly abrogated the inhibition of PCa cell proliferation induced by miR-195 (Fig. 1a). As expected, overexpression of BCOX1 significantly reversed the suppression of PCa cell migration and invasion induced by miR-195 (Fig. 1b, c, d, e).BCOX1 knockdown can result in similar results induced by miR-195 expression in PCa cells. BCOX1 expression was significantly decreased by miR-195 and BCOX1 knockdown in PCa cells. As shown colony formation assays, we identified that both miR-195 and BCOX1 knockdown caused a comparable suppression of cell growth (Fig. 1a). As for migration and invasion, BCOX1 knockdown can mimic the suppression of PCa cell migration and invasion induced by miR-195 (Fig. 1b, c, d, e). Collectively, above results showed that miR-195 inhibited PCa cell proliferation, migration and invasion via the inhibition of BCOX1.

### Forced expression of miR-195 inhibits PCa cell growth and metastasis *in vivo*

**Fig. 4:**
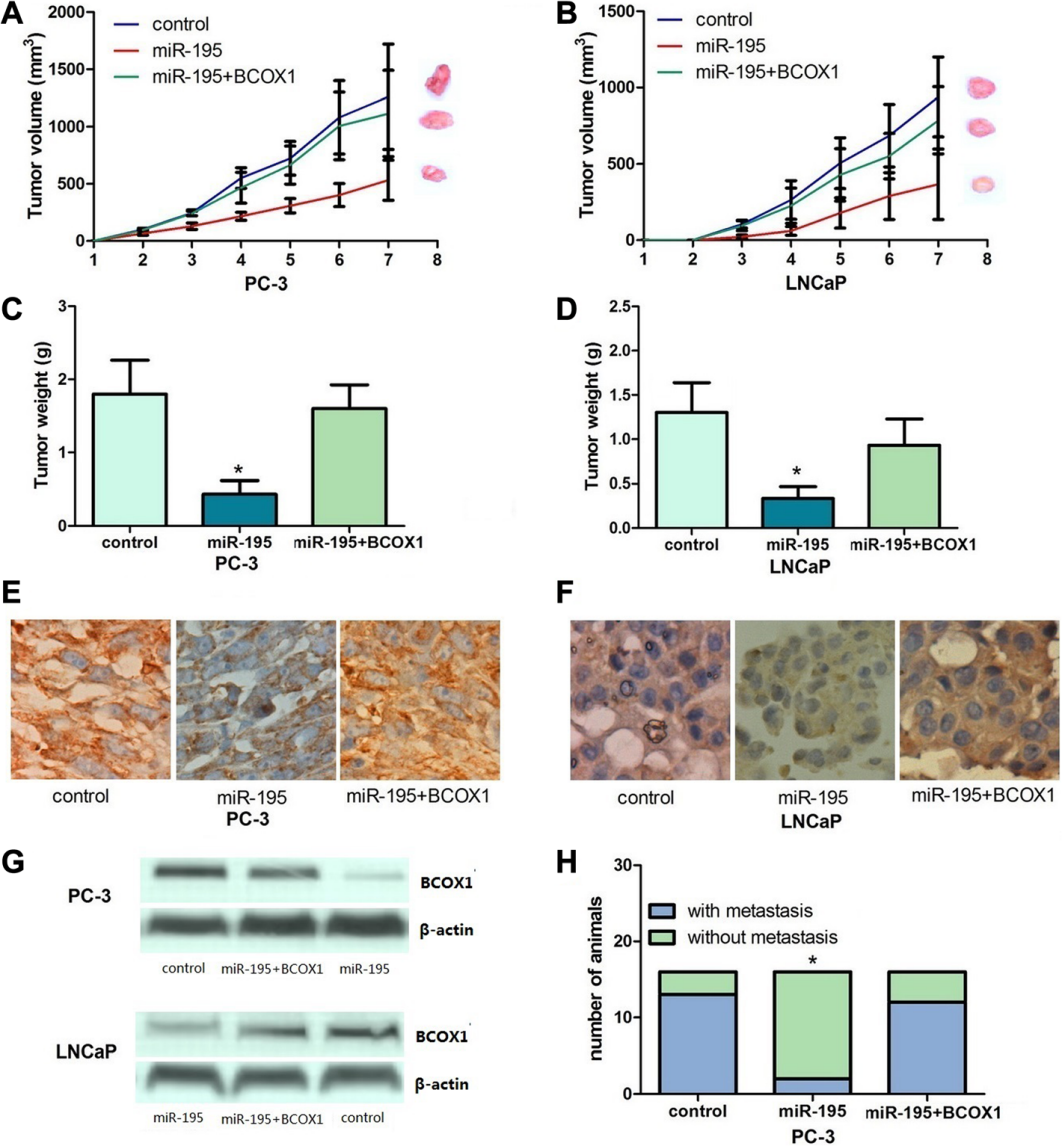
Ectopic expression of miR-195 inhibits tumor growth and metastasis via targeting BCOX1 *in vivo*. Restoration of BCOX1 significantly reversed the suppression of tumor growth and metastasis induced by miR-195. **a** and **b**, Ectopic expression of miR-195 in PCa cells significantly inhibits tumor growth in a mouse xenograft model. **c** and **d**, Tumor weights of corresponding mouse xenograft models. **e** and **f**, BCOX1 expression analysis was performed at protein level by immunochemistry. **g** and **h**, BCOX1 expression analysis was conducted at protein level by western blot. I, miR-195 played an important role in PCa metastasis *in vivo* via targeting BCOX1

To validate the results obtained from in vitro studies, we investigated the in vivo relevance of miR-195-mediated regulation of PCa metastasis by using a PCa xenograft mouse model. Stable PCa cells expressing miR-195 significantly inhibited tumor growth and weight in mice (Fig. 4a, b, c, d) compared with control xenografts indicating that forced expression of miR-195 attenuates tumor growth *in vivo*. Immunohistochemistry and western blots confirmed that xenograft tumors expressing miR-195 showed decreased staining for BCOX1. The results showed attenuated metastasis in the miR-195 expressing PC-3 group compared to the control group. Consistent with this *in vitro* data, forced expression of BCOX1 significantly reversed the inhibition of tumor growth and metastasis induced by miR-195. We did not find any metastases in control or experimental LNCaP xenografts. Above results clearly indicated that miR-195 played an important role in PCa growth and metastasis *in vivo* via targeting BCOX1.

## Discussion

Ample evidence indicates a crucial role for miRNAs in triggering cancer development and metastasis [16–18]. Previous studies indicated that miR-195 play an important role in anti-proliferation and anti-metastasis properties in several types of cancers [19–23]. However, the molecular mechanism of miR-195 deregulation in PCa remains elusive, so our team aimed to study the biological functions and underlying molecular mechanisms of miR-195 in PCa. Although considerable advances in diagnosis and adjuvant therapy of PCa have been made, many patients with PCa will develop metastases, the overall survival rate has not been improved markedly [24–29]. Although several clinical factors, such as PSA and Gleason score, may provide some prognostic utility in the treatment settings, there are currently no definitive clinical methods that can reliably predict the responses to clinical therapies for patients with PCa [30–35]. Therefore, there is an urgent need for prognostic biomarkers to strengthen the efficiency of early diagnosis and to improve the therapeutic strategies of PCa [36–40]. We found that miR-195 was significantly decreased in PCa and that low miR-195 expression was an independent predictor for the poor outcome of patients with PCa. We identified that miR-195 can inhibit PCa cell proliferation, invasion and migration in vitro, and suppress PCa growth and metastasis in vivo by directly targeting BCOX1. Taken together, above data indicated that miR-195 played critical roles in PCa progression.

It is well known that PSA can act as the most common marker for following the course of PCa [36, 41–43]. Previous study indicated that miR-188-5p can also be used as a useful biomarker for following PCa, and it can directly regulated the LAPTM4B in PCa [36]. It well known that the course of PCa progression and clinical outcomes of patients with PCa can differ even in patients with the same PSA status [36]. Therefore, there is urgency to find more sensitive biomarkers for following the course of PCa and improvement of PCa prognosis. In order to study this, we investigate miR-195 expression and its association with the clinicopathological factors of PCa patients. The results indicated that low miR-195 expression can serve as a useful biomarker in identifying poor outcomes for patients with PCa. Collectively, low miR-195 expression was an independent prognostic biomarker for worse survival. Further large-scale cohort studies may be needed to confirm whether miR-195 is an effective prognostic biomarker.

miR-195 was identified to be decreased in metastatic PCa, and its low expression was associated with poor prognosis in PCa patients, which strongly indicates a potential role of miR-195 in suppression of PCa. These observations suggested that decreased miR-195 expression in PCa may facilitate development of an invasive/metastatic phenotype. We performed a computational search for the potential targets for miR-195. BCOX1 was identified as a potential target for miR-195. BCOX1 gene was highly homologous to hypothetical gene KIAA0100, which maps to chromosome 17q11.2 and was first reported in breast cancer [44]. The BCOX1 was predicted to encode a 222-amino acid BCOX1 protein, with an estimated molecular mass of 24.9 kD [45]. Previous studies indicated that BCOX1 overexpression can promote the recurrence and progression of triple negative breast cancer. BCOX1 overexpression was also a valuable prognostic marker for evaluating the survival of triple negative breast cancer patients [45]. Microarray data showed that BCOX1 was significantly increased in metastatic PCa tissues relative to primary PCa tissues and normal prostate tissues. These data also showed that BCOX1 is significantly overexpressed in patients with lymph node metastasis, higher Gleason score and BCR compared with patients without lymph node metastasis, BCR, and with lower Gleason score, respectively. Above observations indicated that the increased BCOX1 is associated with PCa progression. The miR-195 expression levels were found to be correlated inversely with BCOX1 mRNA expression levels. We further identified that miR-195 can negatively regulate BCOX1 expression. These results indicated that BCOX1 was a direct target of miR-195.

Forced expression of miR-195 significantly inhibited the proliferation, migration and invasion of PCa cells *in vitro*, and tumor growth and metastasis *in vivo*. Our data indicated that PCa cells became less aggressive and invasive after transfected with the miR-195 expression construct, indicating that the miR-195 may serve as a metastasis suppressor in PCa. Our data further indicated that the metastasis-suppressive miR-195 can target BCOX1 and that silencing of BCOX1 significantly inhibits the proliferation, migration and invasion of PCa cells. In addition, overexpression of BCOX1 can significantly reverse the inhibitory effects of miR-195. Our study also confirmed the association between mir-195 and BCR in a recently published paper [19]. Previous study results also indicated that miR-195 was a critical tumor suppressor in PCa progression which is similar with our study results [19]. These observations showed that the increased BCOX1 can drive PCa progression by promote PCa proliferation, migration and invasion. Collectively, our studied indicate that miR-195 inhibits PCa progression by directly silencing BCOX1.

In summary, this is the first report unveiled that miR-195 can serve as a novel player with metastasis suppressor functions in PCa progression and metastasis. These findings provide new insight into the molecular pathogenesis of PCa and implicate miR-195 as a potential prognostic biomarker and therapeutic target of PCa.

## Additional files

Additional file 1: Figure S1. miR-195 is under-expressed in metastasis PCa. miR-195 expression was decreased in metastatic PCa compared to primary PCa tissues. (JPEG 117 kb) Additional file 2: Table S1. Clinicopathologic factors and miR-195 expression in 130 PCa patients. (DOC 53 kb) Additional file 3: Table S2. Prognostic value of miR-195 expression for the biochemical recurrence free survival in univariate and multivariate analyses by Cox regression. (DOC 35 kb) Additional file 4: Table S3. Prognostic value of miR-195 expression for the overall survival in univariate and multivariate analyses by Cox regression. (DOC 34 kb)
