# Supplementary figures and images for: MicroRNA-195 suppresses tumor cell proliferation and metastasis by directly targeting BCOX1 in prostate carcinoma
Source: J Exp Clin Cancer Res. 2015 Sep 4;34(1):91. doi: 10.1186/s13046-015-0209-7 (PMC4559360; doi:10.1186/s13046-015-0209-7)

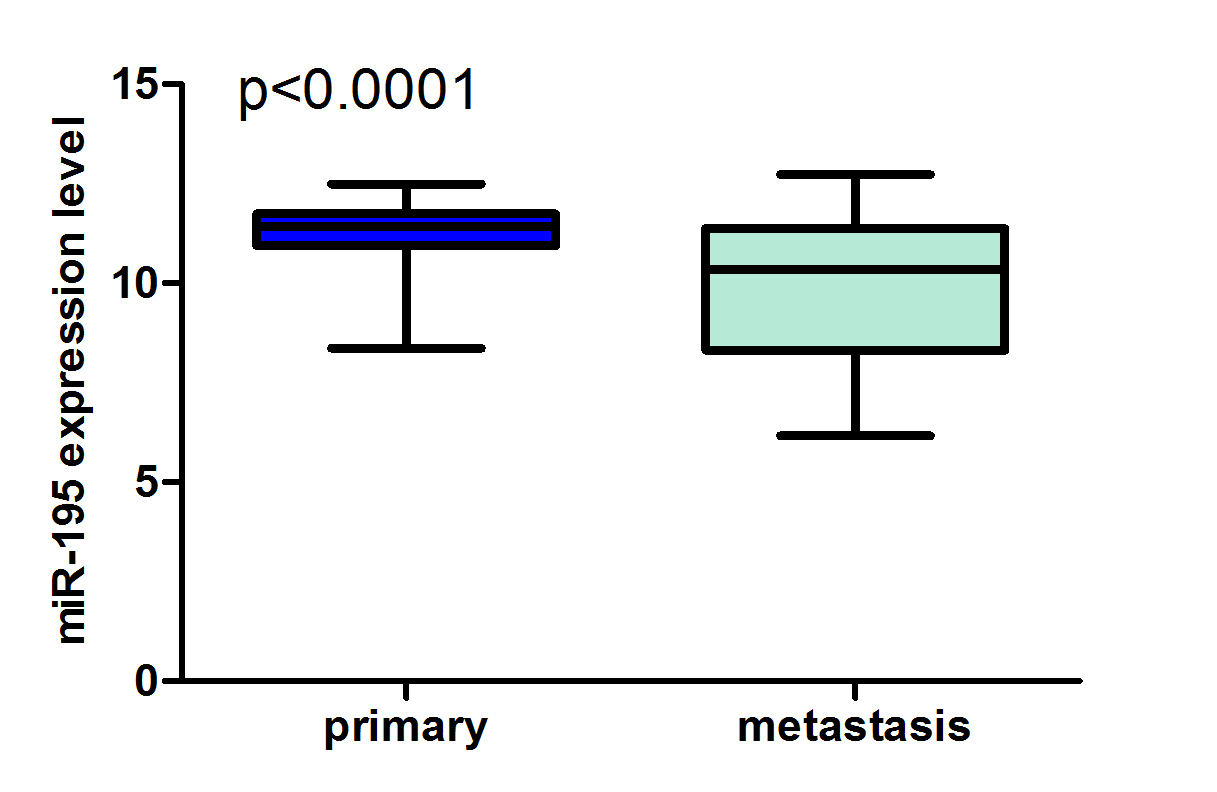

Supplement: Additional file 1: Figure S1. — miR-195 is under-expressed in metastasis PCa. miR-195 expression was decreased in metastatic PCa compared to primary PCa tissues. (JPEG 117 kb) [file 13046_2015_209_MOESM1_ESM.jpg]
