# Supplementary material for: MicroRNA-195 suppresses tumor cell proliferation and metastasis by directly targeting BCOX1 in prostate carcinoma
Source: J Exp Clin Cancer Res. 2015 Sep 4;34(1):91. doi: 10.1186/s13046-015-0209-7 (PMC4559360; doi:10.1186/s13046-015-0209-7)
Supplement: Additional file 2: Table S1. — Clinicopathologic factors and miR-195 expression in 130 PCa patients. (DOC 53 kb) [file 13046_2015_209_MOESM2_ESM.doc]

**Supplementary Table 1: Clinicopathologic factors and miR-195 expression in 140 PCa patients**

|  |  | miR-195 expression | | |  |
| --- | --- | --- | --- | --- | --- |
| Variable | Group | High (n=70) | Low (n=70) | Total | P value |
| Age | ＜70 | 39 | 38 | 77 | 0.865 |
|  | ≥70 | 31 | 32 | 63 |  |
| Lymph node metastasis | Absence | 68 | 56 | 124 | 0.001 |
|  | Presence | 2 | 14 | 16 |  |
| Surgical margin status | Absence | 64 | 62 | 126 | 0.573 |
|  | Presence | 6 | 8 | 14 |  |
| Seminal vesicle invasion | Absence | 63 | 49 | 112 | 0.003 |
|  | Presence | 7 | 21 | 28 |  |
| Clinical stage | T1 | 52 | 33 | 85 | 0.001 |
|  | T2/T3 | 18 | 37 | 55 |  |
| Preoperative PSA | ＜4 | 5 | 1 | 6 | 0.029 |
|  | 4-10 | 25 | 20 | 45 |  |
|  | ＞10 | 30 | 49 | 79 |  |
| Gleason score | ＜7 | 52 | 13 | 65 | ＜0.001 |
|  | 7 | 12 | 22 | 34 |  |
|  | ＞7 | 6 | 35 | 41 |  |
| Angiolymphatic invasion | Absence | 61 | 52 | 113 | 0.054 |
|  | Presence | 9 | 18 | 27 |  |
| Biochemical recurrence | Absence | 62 | 47 | 109 | 0.002 |
|  | Presence | 8 | 23 | 31 |  |
