# Supplementary material for: MicroRNA-195 suppresses tumor cell proliferation and metastasis by directly targeting BCOX1 in prostate carcinoma
Source: J Exp Clin Cancer Res. 2015 Sep 4;34(1):91. doi: 10.1186/s13046-015-0209-7 (PMC4559360; doi:10.1186/s13046-015-0209-7)
Supplement: Additional file 3: Table S2. — Prognostic value of miR-195 expression for the biochemical recurrence free survival in univariate and multivariate analyses by Cox regression. (DOC 35 kb) [file 13046_2015_209_MOESM3_ESM.doc]

**Supplementary Table 2: Prognostic value of miR-195 expression for the biochemical recurrence free survival in univariate and multivariate analyses by Cox regression.**

|  | Univariate analysis | | | Multivariate analysis | | |
| --- | --- | --- | --- | --- | --- | --- |
| Covariant | Exp (B) | 95% CI | P value | Exp (B) | 95% CI | P value |
| miR-195 expression | 5.978 | 2.512-14.223 | <0.001 | 5.958 | 1.182-30.023 | 0.031 |
| Gleason score | 3.477 | 1.436-8.417 | 0.006 | 2.413 | 0.954-6.101 | 0.063 |
| Lymph node metastasis | 4.381 | 1.765-10.872 | 0.001 | 4.066 | 1.708-9.667 | 0.002 |
| Preoperative PSA | 2.038 | 0.858-4.838 | 0.107 |  |  |  |
| Angiolymphatic invasion | 1.079 | 0.802-1.439 | 0.526 |  |  |  |
| Surgical margin status | 2.830 | 0.796-10.064 | 0.108 |  |  |  |
| PCa Stage | 3.411 | 0.427-27.254 | 0.247 |  |  |  |
| Seminal vesicle invasion | 3.280 | 0.944-11.394 | 0.062 |  |  |  |
| Age | 0.912 | 0.386-2.155 | 0.834 |  |  |  |
