# Supplementary material for: MicroRNA-195 suppresses tumor cell proliferation and metastasis by directly targeting BCOX1 in prostate carcinoma
Source: J Exp Clin Cancer Res. 2015 Sep 4;34(1):91. doi: 10.1186/s13046-015-0209-7 (PMC4559360; doi:10.1186/s13046-015-0209-7)
Supplement: Additional file 4: Table S3. — Prognostic value of miR-195 expression for the overall survival in univariate and multivariate analyses by Cox regression. (DOC 34 kb) [file 13046_2015_209_MOESM4_ESM.doc]

**Supplementary Table 3: Prognostic value of miR-195 expression for the overall survival in univariate and multivariate analyses by Cox regression**

|  | Univariate analysis | | | Multivariate analysis | | |
| --- | --- | --- | --- | --- | --- | --- |
| Covariant | Exp (B) | 95% CI | P value | Exp (B) | 95% CI | P value |
| miR-195 expression | 4.195 | 1.402-12.554 | 0.010 | 4.462 | 1.353-14.722 | 0.014 |
| Surgical margin status | 2.515 | 0.865-7.312 | 0.090 |  |  |  |
| Preoperative PSA | 2.288 | 0.763-6.857 | 0.140 |  |  |  |
| PCa Stage | 3.008 | 1.008-8.981 | 0.048 | 3.583 | 1.085-11.829 | 0.036 |
| Age | 1.032 | 0.964-1.104 | 0.367 |  |  |  |
| Angiolymphatic invasion | 1.053 | 0.344-3.220 | 0.928 |  |  |  |
| Gleason score | 3.894 | 1.305-11.621 | 0.015 | 3.420 | 1.041-11.228 | 0.043 |
| Lymph node metastasis | 3.150 | 0.404-24.562 | 0.274 |  |  |  |
| Seminal vesicle invasion | 2.866 | 0.786-10.459 | 0.111 |  |  |  |
